# IGFBP-1 is associated with IRS signaling upregulation and contributes to metabolic recovery post-Roux-en-Y bypass

**DOI:** 10.3389/fendo.2026.1859266

**Published:** 2026-07-16

**Authors:** Tengfei Qi, Ru Ji, Liangping Wu, Jipei He, Hongbin Zhang, Zhengyong Xie

**Affiliations:** 1 Department of General Surgery, The General Hospital of Southern Theater Command, Guangzhou, China; 2 Department of Hepatobiliary Surgery and Liver Transplantation Center, General Hospital of Southern Theater Command, Guangzhou, China; 3 Jinshazhou Hospital of Guangzhou University of Chinese Medicine, Guangzhou, China; 4 Department of Basic Medical Research, General Hospital of Southern Theater Command, Guangzhou, China; 5 Southern Medical University School of Laboratory Medicine and Biotechnology, Guangzhou, China; 6 The First School of Clinical Medicine, Southern Medical University, Guangzhou, China

**Keywords:** glucose metabolism, insulin resistance, insulin-like growth factor binding protein, integrin, Roux-en-Y gastric bypass

## Abstract

**Objective:**

To investigate whether increased IGFBP-1 following Roux-en-Y gastric bypass (RYGB) surgery is associated with upregulation of IRS-1 and its downstream PI3K/AKT/GSK-3β signaling components, thereby contributing to the glucose-lowering mechanism of metabolic surgery.

**Methods:**

Wild-type C57BL/6 mice (WT) and C57BL/6-Igfbpem1 IGFBP-1 knockout mice (KO) were used, along with an induced diabetes model (high-fat diet for 6 weeks followed by low-dose streptozotocin for 5 days prior to surgery). Preoperatively and at 1 to 8 weeks postoperatively, body weight, fasting blood glucose, and Homeostatic Model Assessment of Insulin Resistance (HOMA-IR, calculated from fasting glucose and insulin at week 8) were assessed. Plasma samples were collected and stored at -80 °C. Mice were euthanized at eight weeks postoperatively, and tissues were collected. ELISA was used to measure plasma IGFBP-1, IRS-1, PI3K, AKT, GSK-3β. Real-time PCR was performed to assess mRNA expression of these targets in multiple tissues. This work is reported in accordance with the ARRIVE guidelines.

**Results:**

Following RYGB, body weight, fasting blood glucose, and HOMA-IR improved significantly. IGFBP-1 levels were significantly elevated post-surgery, along with marked upregulation of IRS-1, PI3K, and AKT, and downregulation of GSK-3β in WT mice. These changes were absent in KO mice. Histomorphometric analysis demonstrated that RYGB reversed multi-organ pathological changes (cardiomyocyte hypertrophy, hepatic steatosis, glomerular enlargement, adipocyte hypertrophy) in WT mice but only partially in KO mice.

**Conclusion:**

Metabolic surgery upregulates IGFBP-1 expression. Elevated IGFBP-1 is associated with upregulation of the IRS/PI3K/AKT/GSK-3β signaling axis and improved insulin sensitivity. IGFBP-1 is necessary for achieving complete normalization of glucose homeostasis and multi-tissue repair after RYGB.

## 1 Introduction

Diabetes mellitus and obesity represent a significant global public health challenge, with an increase in the age-standardized prevalence of diabetes mellitus among adults in every country since 1980 (1). In 1995, Pories first demonstrated that gastric bypass could not only control weight but also effectively manage non-insulin-dependent diabetes in the long term (2). Type 2 diabetes develops as a result of insulin resistance coupled with progressive pancreatic β-cell dysfunction (3, 4). Several studies, both domestic and international, have demonstrated that metabolic surgery significantly reduces insulin resistance (5–9). However, the precise mechanisms by which metabolic Surgery influences insulin sensitivity remain inadequately understood. Currently, several predominant theories exist to explain the glucose-lowering effects of metabolic surgery, including the foregut hypothesis (which proposes that exclusion of the duodenum and proximal jejunum removes a putative anti-incretin signal), the hindgut hypothesis (which emphasizes enhanced secretion of GLP-1 and PYY from the distal intestine), the gastric center theory, and the gut-brain axis theory (10–12). However, most studies focus on comparing changes in the expression of specific gastrointestinal hormones before and after metabolic surgery, which fails to capture the comprehensive glucose-lowering mechanisms involved. While these gut−centric hypotheses have advanced our understanding, a comprehensive picture of the glucose−lowering mechanisms remains incomplete. A liver−derived endocrine component may represent a complementary pathway, as suggested by our genomic screening described below.

Our team previously performed genomic profiling of blood samples from patients with obesity before and six months after metabolic surgery (13). This analysis revealed significant alterations in multiple signaling pathways, including the insulin/IGFs/PKB pathway, the MAPK pathway, and the PI3K−AKT pathway. Among these, the IGF signaling pathway showed the most prominent changes, and we therefore focused on this axis (**Supplementary Figure 1**). Subsequent ELISA measurements indicated that plasma IGF−1 levels did not change significantly after surgery. However, we observed a marked postoperative increase in the IGF−1 binding protein, IGFBP−1 (**Supplementary Figure 2**). To confirm that this elevation was specifically attributable to the surgical intervention, we analyzed plasma samples from three groups: (1) 10 patients with obesity who underwent Roux−en−Y gastric bypass (RYGB) surgery, with samples collected three days before and six months after surgery; (2) 30 normal−weight individuals; and (3) 27 patients with obesity who did not receive metabolic surgery. The results showed that preoperative IGFBP−1 levels did not differ among the groups, but post−RYGB IGFBP−1 levels were significantly higher than those in the non−operated obese control group (**Supplementary Figure 3**). While other IGFBPs, particularly IGFBP−2, have been reported to increase following metabolic surgery and associate with improved insulin sensitivity (14), our candidate−driven approach was guided by a rigorous clinical screening strategy that consistently identified IGFBP−1 as the most dynamically and specifically altered component within the IGF system. We therefore prioritized mechanistic validation of IGFBP−1 as our primary candidate. Moreover, IGFBP−1 uniquely contains an RGD (Arg−Gly−Asp) integrin−binding motif, suggesting a potential direct signaling capacity that distinguishes it from other IGFBPs and merits dedicated mechanistic investigation (15, 16). Based on these clinical observations and a targeted literature review (15, 17–19), we hypothesize that IGFBP−1 plays a critical regulatory role within the IGF system after metabolic surgery. The insulin/IGF−1−induced elevation of insulin receptor substrate (IRS) and its downstream PI3K/AKT signaling pathway is a well−established mechanism underlying the metabolic benefits of bariatric surgery. Therefore, we propose that IGFBP−1 may be associated with the upregulation of IRS and the PI3K/AKT/GSK−3β signaling axis, thereby contributing to the glucose−lowering effect of RYGB.

## 2 Experimental materials

### 2.1 Experimental animals

SPF-grade C57BL/6-Igfbpem1 homozygous mice (3 males, 6 females, aged 8 weeks, weight 23–26 g) were procured from Shanghai Model Organisms Center, Inc. (license SCXK (Shanghai) 2018-0007). Animals were housed in the Animal Experiment Center at 18-22 °C, humidity 55-65%, with ad libitum access to water and standard diet. Breeding was performed to obtain sufficient male homozygotes, confirmed by tail genotyping using agarose gel electrophoresis. Age- and weight-matched SPF male C57BL/6 mice were obtained as wild-type (WT) controls. Male mice were used to minimize potential variability due to estrous cycle (20, 21). All animal experiments were approved by the Ethics Committee for Animal Experimentation of Zhujiang Hospital, Southern Medical University (License Nos. LAEC-2021–070 and LAEC-2022-141), conducted in compliance with the Laboratory Animal Management Regulations and reported in accordance with the ARRIVE guidelines (22).

### 2.2 Data and methods

#### 2.2.1 Study groups

Mice were acclimated for one week (starting at 7 weeks of age) on a standard diet. Subsequently, mice were randomly assigned to groups (n = 6 per group): Wild-type Blank Control (WT-CON), Wild-type SHAM (WT-SHAM), Wild-type RYGB (WT-RYGB), IGFBP-1 Knockout Blank Control (KO-CON), IGFBP-1 Knockout SHAM (KO-SHAM), and IGFBP-1 Knockout RYGB (KO-RYGB).

#### 2.2.2 Interventions

All mice were maintained on a standard diet until 8 weeks of age. From 8 to 14 weeks of age, mice in the SHAM and RYGB groups were switched to a high-fat diet (HFD, 60% kcal from fat, Product #HF60, Dyets, Inc., Bethlehem, PA, USA) to induce obesity and insulin resistance. The CON groups remained on standard diet throughout.

At 14 weeks of age (after 6 weeks of HFD), diabetes was induced by intraperitoneal injection of streptozotocin (STZ, 40 mg/kg, MBCHEM, protected from light) daily for 5 consecutive days. Nine days after the final injection, fasting blood glucose (FBG) was measured for three consecutive days. Mice with FBG >16.7 mmol/L were considered to have successfully developed a type 2 diabetes mellitus model (23, 24). Mice meeting the glucose criteria were randomly assigned to SHAM or RYGB groups (n=6 per group). The SHAM group underwent a sham operation, while the RYGB group underwent Roux-en-Y gastric bypass surgery as previously described (25). Briefly, under Zoletil 50 (20 mg/kg) and Xylazine (20 mg/kg) anesthesia (26), a proximal gastric pouch (10% of total gastric volume) was created, and the small intestine was transected 4 cm distal to the ligament of Treitz. The distal segment was anastomosed to the gastric pouch, and the proximal segment was anastomosed to the small intestine 6 cm distal to the gastrojejunostomy. SHAM surgery involved a gastrotomy and intestinal incision closed with 9–0 sutures to replicate surgical trauma without anatomical alteration.

Body weight and FBG were measured weekly. At 8 weeks post-surgery, after an overnight fast, blood samples were collected from the orbital venous plexus. Fasting serum insulin levels were measured using a mouse insulin ELISA kit. FBG was measured simultaneously using a glucometer. The Homeostatic Model Assessment for Insulin Resistance (HOMA-IR) was calculated as: HOMA-IR = [FBG (mmol/L) × fasting insulin (mIU/L)]/22.5.

At 8 weeks post-surgery, mice were euthanized by isoflurane overdose (5% in 100% oxygen, 1 L/min). Loss of consciousness was confirmed by absence of pedal reflex. Blood was collected via cardiac puncture, and plasma was isolated and stored at -80 °C. Death was confirmed by cessation of heartbeat. Tissues were harvested for histology or snap-frozen for RNA analysis. This method is consistent with the American Veterinary Medical Association (AVMA) Guidelines for the Euthanasia of Animals and was approved by the institutional animal care and use committee (License Nos. LAEC-2021–070 and LAEC-2022-141).

#### 2.2.3 ELISA measurements

Plasma levels of IGFBP-1 (MEIMIAN, MM-0179M1), IRS-1 (MEIMIAN, MM-44840M2), PI3K (MEIMIAN, MM-0428M1), AKT (MEIMIAN, MM-20286M1), and GSK-3β (MEIMIAN, MM-0683M1) were measured using commercially available ELISA kits according to the manufacturer instructions. All samples were run in duplicate.

#### 2.2.4 Real-time PCR

Total RNA was extracted from tissues using TRIzol reagent. Reverse transcription was performed using a PrimeScript RT reagent kit. Real-time PCR was carried out using SYBR Green master mix on a CFX96 system. Primer sequences are listed in **Table 1**. Relative mRNA expression was calculated using the 2^-ΔΔCt method with GAPDH as the internal control.

**Table 1:** Experiment-specific primer information.

Target gene	Primer name	Sequence
	mus-GAPDH-R	GATGCAGGGATGATGTTCTGGG
IGFBP-1	mus-IGFBP-1-F	CCCAACAGAAAGCAGGAGATGA
	mus-IGFBP-1-R	TTCTCCATCCAGGGATGTCTCA
IRS-1	mus-IRS-1-F	TGTCACCCAGTGGTAGTTGCTC
	mus-IRS-1-R	CTCTCAACAGGAGGTTTGGCATG
PI3K	mus-PI3K-F	CAAACCACCCAAGCCCACTACT
	mus-PI3K-R	CCATCAGCAGTGTCTCGGAGTT
AKT	mus-AKT-F	GGACTACTTGCACTCCGAGAAG
	mus-AKT-R	CATAGTGGCACCGTCCTTGATC
GSK-3β	mus-GSK-3β-F	GAGCCACTGATTACACGTCCAG
	mus-GSK-3β-R	CCAACTGATCCACACCACTGTC

#### 2.2.5 Histological and morphometric analysis

Tissues were fixed in 4% paraformaldehyde, embedded in paraffin, sectioned at 4 µm, and stained with hematoxylin and eosin (H&E). Liver sections were also stained with Oil Red O for lipid assessment. For each tissue, quantitative morphometric analysis was performed by an investigator blinded to group allocation using ImageJ software (NIH, USA). Detailed methodology for each tissue is provided in the Results section. For each animal, at least three randomly selected, non-overlapping fields were analyzed, and a minimum of 50 cells/structures were measured per tissue (n=6 animals per group).

#### 2.2.6 Statistical processing

**Figure 1:**
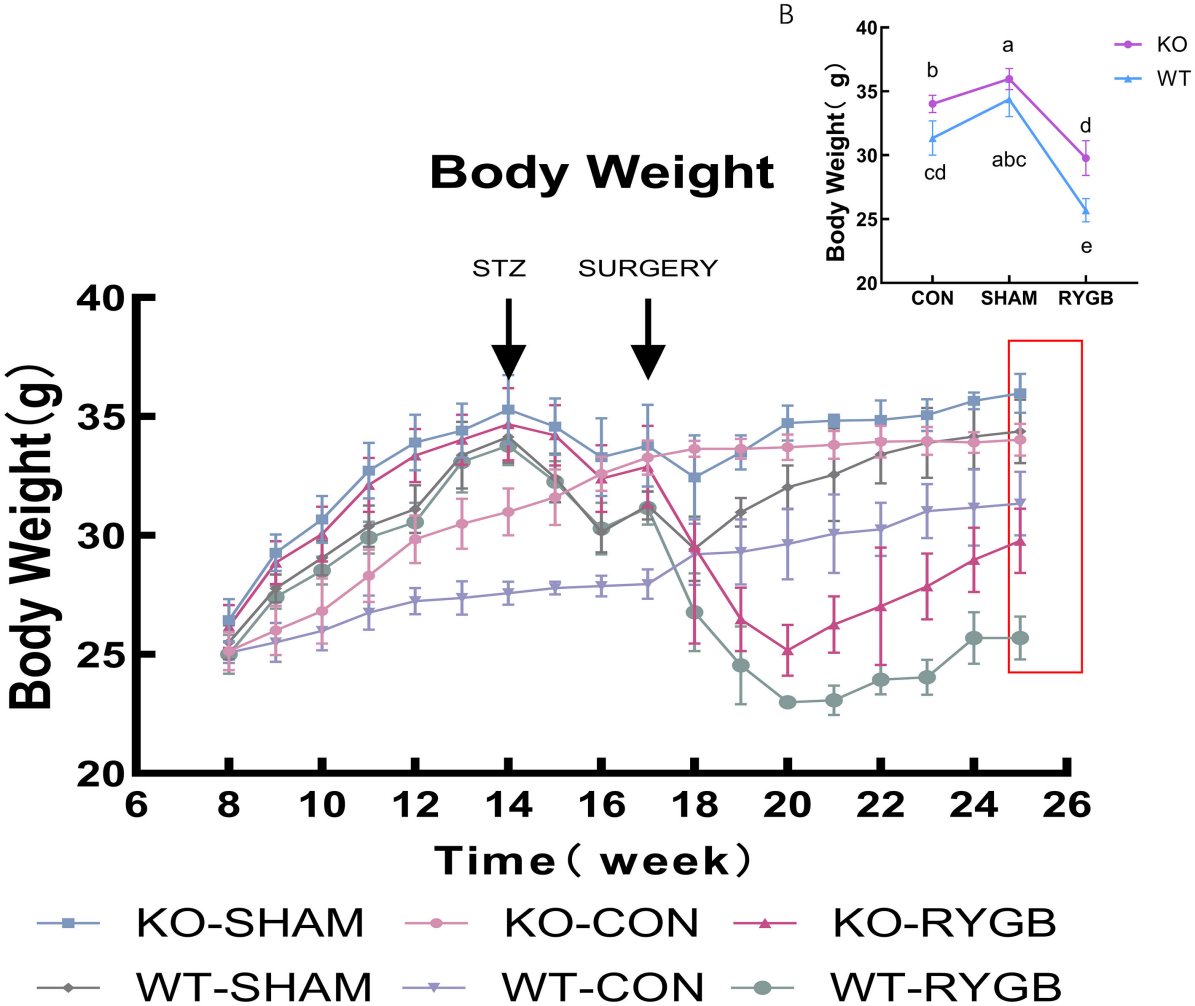
Body weight changes before and after RYGB surgery in WT and KO mice. **(A)** Weekly body weight measurements from 8 to 25 weeks of age. Mice in the SHAM and RYGB groups were fed a high-fat diet from weeks 8–14 and received streptozotocin (STZ) at week 14 to induce diabetes. RYGB or SHAM surgery was performed at week 17 (indicated by arrow). CON groups remained on standard diet throughout. Data are shown as mean ± SD, n = 6 per group. **(B)** Body weight at 8 weeks post-surgery (week 25). Two-way ANOVA (factors: genotype and surgery) revealed significant main effects of surgery (F = 56.6, P < 0.001) and genotype (F = 139.2, P < 0.001), as well as a significant interaction (F = 3.779, P < 0.05). Tukey’s post-hoc test was used for multiple comparisons. *P < 0.05, **P < 0.01, ***P < 0.001.

**Figure 2:**
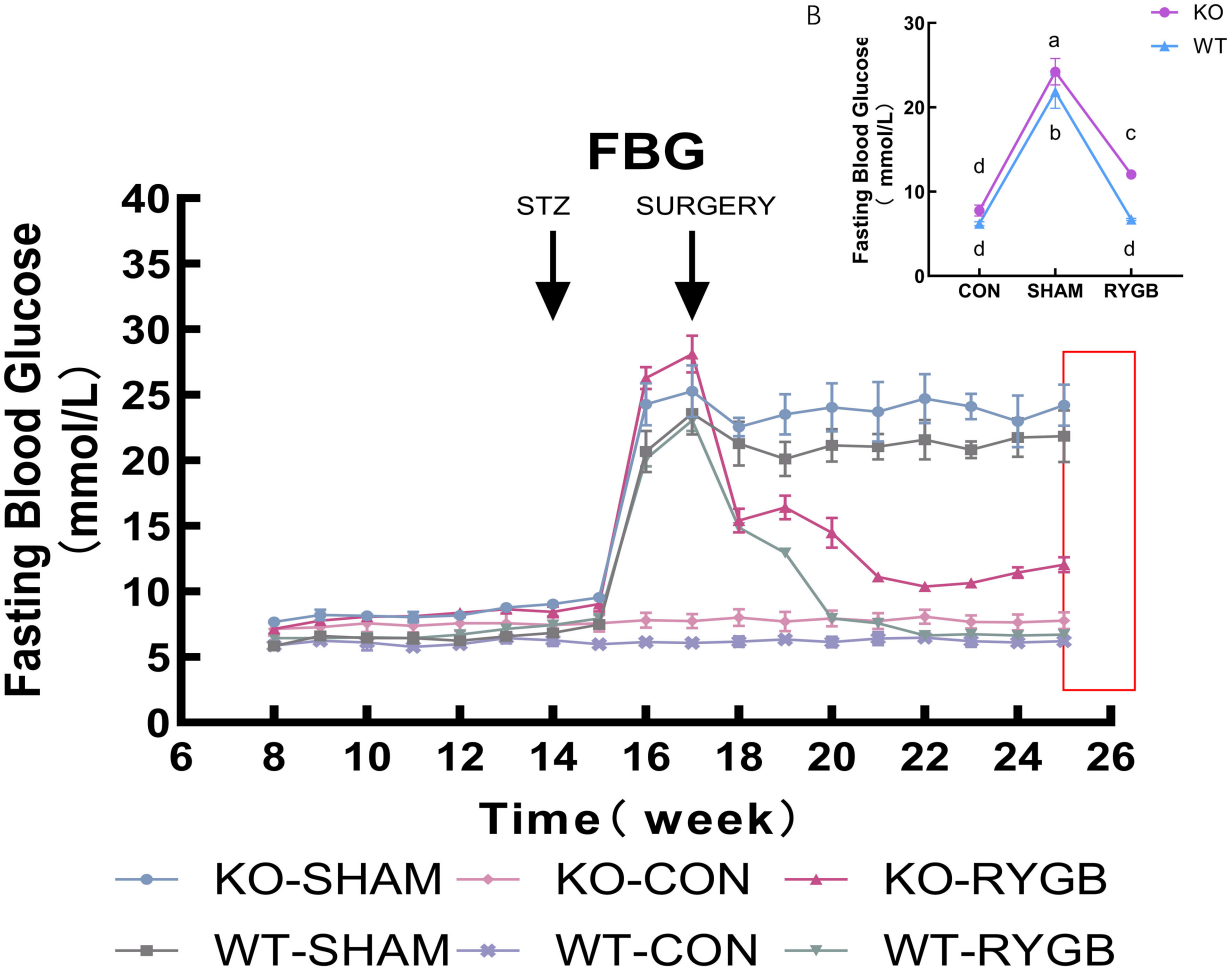
Fasting blood glucose (FBG) before and after RYGB surgery in WT and KO mice. **(A)** Weekly FBG measurements from 8 to 25 weeks of age. Diabetes was induced by HFD + STZ at week 14, and RYGB or SHAM surgery was performed at week 17 (arrow). CON groups remained on standard diet. Data are shown as mean ± SD, n = 6 per group. **(B)** FBG at 8 weeks post-surgery (week 25). Two-way ANOVA (factors: genotype and surgery) revealed significant main effects of surgery (F = 122, P < 0.001) and genotype (F = 472, P < 0.001), as well as a significant interaction (F = 20.91, P < 0.01). Tukey’s post-hoc test was used for multiple comparisons. *P < 0.05, **P < 0.01, ***P < 0.001.

In this study, Microsoft Excel was used for data recording, and SPSS 26.0 was employed for data analysis. All numerical data were subjected to normality testing. If the data followed a normal distribution, they were expressed as mean ± standard deviation (mean ± SD); if the data deviated from normality, they were expressed as median (Q1, Q3). When numerical data followed a normal distribution, two-group comparisons were conducted using t-tests, multiple comparisons were made using ANOVA, and pairwise comparisons were performed using the LSD method. For data that deviated from normality, nonparametric statistical methods were employed. For categorical data, the frequency of each category was reported, and the chi-square test was used to compare the categorical data across groups. A p-value of < 0.05 was considered statistically significant (two-sided test). To present the results more intuitively, graphs and charts were created, and data were visualized using GraphPad Prism 9 software. The specific comparisons were designed to test our primary hypotheses. To assess the effect of RYGB surgery within each genetic background, data from Wild-type (WT) and IGFBP-1 Knockout (KO) mice were analyzed separately using one-way ANOVA (comparing Control, SHAM, and RYGB groups), followed by *post-hoc* tests where appropriate. To directly test our central hypothesis that the metabolic outcome of surgery is modulated by IGFBP-1, planned comparisons between genotypes (WT compared with. KO) were conducted using independent t-tests within each surgical treatment group (e.g., WT-RYGB compared with. KO-RYGB). For the postoperative week 8 data in **Figure 1** and **2**, two−way ANOVA (factors: genotype and surgery) was performed, followed by Tukey’s post−hoc test for multiple comparisons. This comparison directly tests for a genotype-by-surgery interaction effect on the outcome. This focused analytical strategy allows for a direct assessment of genotype-dependent differences in response to identical interventions.

## 3 Results

### 3.1 Effect of RYGB surgery on body weight in mice modeling type 2 diabetes mellitus

As shown in **Figure 1A**, control groups (KO-CON and WT-CON) maintained stable weight gain throughout the observation period. During the baseline phase (weeks 8-14), surgical and SHAM groups exhibited significantly greater weight gain than controls (F-values: 10.35 and 6.262 for KO and WT comparisons, respectively, P < 0.001), with no preoperative differences between surgical subgroups. Following STZ administration, transient weight loss occurred in all intervention groups, stabilizing within one week.

RYGB surgery induced sustained weight reduction relative to SHAM controls in both genotypes (WT-RYGB vs. WT-SHAM: P < 0.001; KO-RYGB vs. KO-SHAM: P < 0.001), with final body weights significantly lower than CON groups (WT-RYGB vs. WT-CON: P < 0.001; KO-RYGB vs. KO-CON: P < 0.001). Notably, WT-CON animals maintained lower body weights than WT-SHAM (P < 0.001), whereas no such difference existed in KO groups (KO-SHAM vs. KO-CON: P > 0.05), suggesting IGFBP-1 deficiency alters this response. Genotype main effect analysis confirmed higher body weights in KO mice across all cohorts: RYGB (t = 2.066, P = 0.0465), SHAM (t = 2.470, P = 0.0187), and CON (t = 3.584, P = 0.0010).

Two-way ANOVA on body weight at 8 weeks post-surgery revealed a significant main effect of surgery (F = 56.6, P < 0.001, **Figure 1B**), a significant main effect of genotype (F = 139.2, P < 0.001), and a significant interaction between surgery and genotype (F = 3.779, P < 0.05). *Post-hoc* comparisons showed that although RYGB significantly reduced body weight in both genotypes, the final body weight of KO-RYGB mice remained significantly higher than that of WT-RYGB mice (P < 0.05).

### 3.2 Effects of RYGB on general condition and fasting blood glucose in mice modeled with type 2 diabetes mellitus

All control groups maintained normal FBG throughout. Following STZ, both surgical and SHAM groups developed hyperglycemia (>16.7 mmol/L). Postoperatively, RYGB groups achieved significant FBG reduction compared to SHAM groups (P < 0.001), with WT-RYGB reaching FBG levels comparable to WT-CON from weeks 22-25 (P > 0.05), whereas KO-RYGB remained significantly elevated compared to KO-CON throughout (P < 0.05, **Figure 2A**). Genotype main effect analysis confirmed higher FBG in KO mice across all groups (RYGB: t = 6.347, P < 0.001; SHAM: t = 12.96, P < 0.001; CON: t = 28.86, P < 0.001).

Two-way ANOVA on FBG at 8 weeks post-surgery (**Figure 2B**) showed a significant main effect of surgery (F = 122, P < 0.001), genotype (F = 472, P < 0.001), and a significant interaction (F = 20.91, P < 0.01), indicating that IGFBP-1 expression is, at least in part, necessary for the full glucose-lowering efficacy of RYGB.

### 3.3 Effect of RYGB on the glycemic response to insulin in mice modeled with type 2 diabetes mellitus

**Figure 3:**
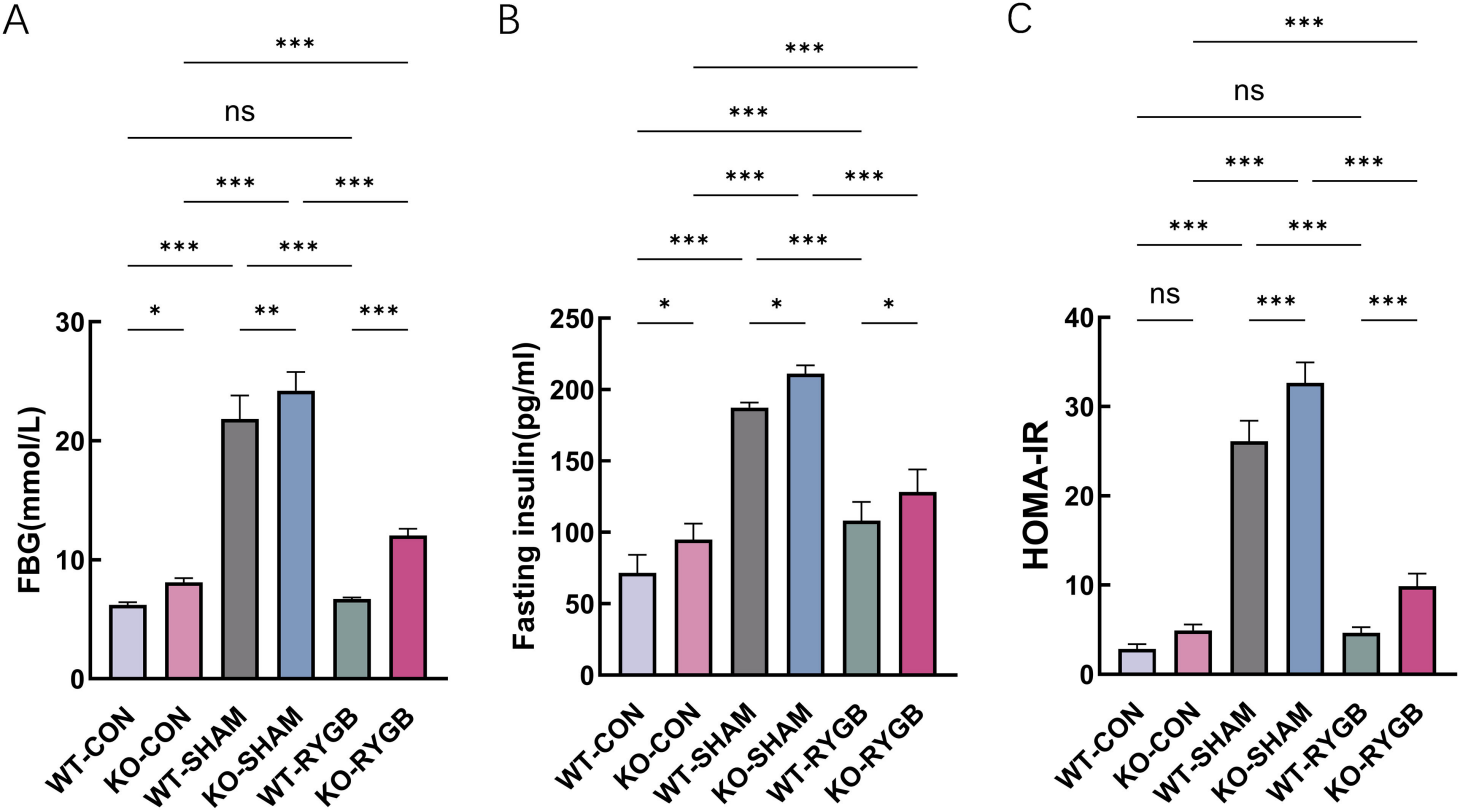
HOMA-IR index at 8 weeks post-surgery. Homeostatic Model Assessment of Insulin Resistance (HOMA−IR) was calculated from fasting blood glucose and fasting insulin levels at 8 weeks after RYGB or SHAM surgery. Data are shown as mean ± SD (n = 6 per group). Two−way ANOVA (factors: genotype and surgery) revealed a significant main effect of surgery (F = 425, P < 0.001) and genotype (not shown), as well as a significant interaction between surgery and genotype (F = 18.43, P < 0.001). Tukey’s post−hoc test was used for multiple comparisons. *P < 0.05, **P < 0.01, ***P < 0.001.

To assess insulin resistance, we calculated HOMA-IR at 8 weeks post-surgery (**Figure 3**). SHAM-operated mice (WT-SHAM and KO-SHAM) exhibited significantly higher HOMA-IR values compared to their respective CON groups (F = 425, P < 0.001). RYGB surgery resulted in a marked reduction in HOMA-IR in both WT-RYGB and KO-RYGB mice compared to SHAM controls (P < 0.001). Critically, the HOMA-IR of WT-RYGB mice was fully normalized, showing no significant difference from WT-CON mice (P > 0.05). In contrast, KO-RYGB mice remained significantly more insulin resistant than KO-CON mice (P < 0.05). Two-way ANOVA revealed a significant interaction between surgery and genotype (F = 18.43, P < 0.001), demonstrating that IGFBP-1 is required for the complete normalization of insulin sensitivity after RYGB.

### 3.4 Effect of RYGB on histopathology of organs in a mouse model of type 2 diabetes mellitus

**Figure 4:**
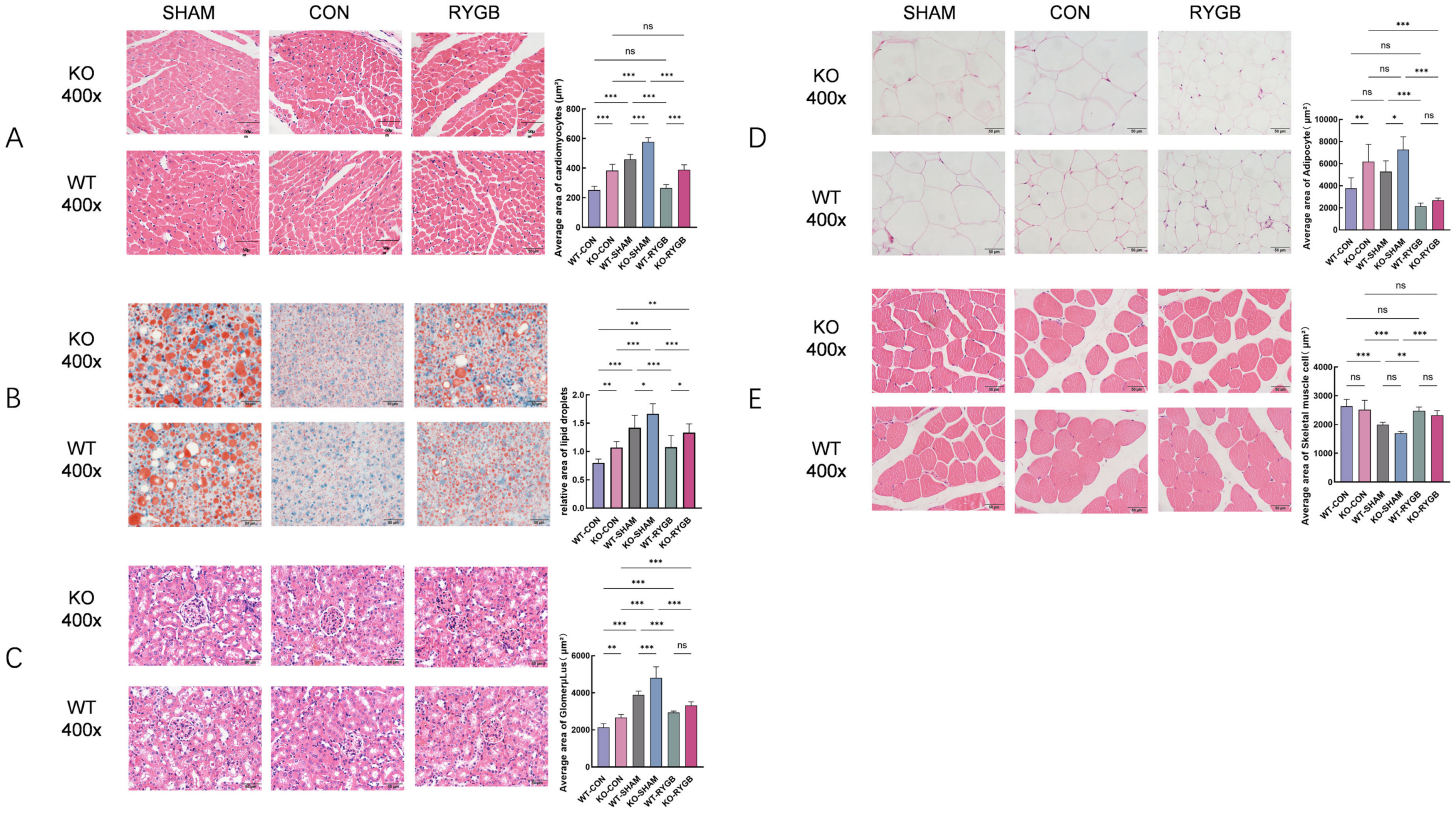
Histopathological analysis of multiple organs in WT and IGFBP-1 KO mice after RYGB surgery. Representative H&E−stained sections of **(A)** heart, **(B)** liver (with Oil Red O staining for lipid), **(C)** kidney, **(D)** inguinal adipose tissue, **(E)** skeletal muscle from WT and IGFBP−1 KO mice at 8 weeks post−surgery. All scale bars = 50 µm. Quantitative morphometric analyses were performed for each organ as described in the Results section. Data are shown as mean ± SD (n = 6 per group). Statistical comparisons were made using one−way ANOVA followed by Tukey’s post−hoc test (within each genotype) or independent t−tests (between genotypes for the same surgical condition). *P < 0.05, **P < 0.01, ***P < 0.001.

Quantitative histomorphometry was performed on H&E−stained sections of multiple organs (heart, liver, pancreas, kidney, inguinal adipose tissue, and skeletal muscle), with all images consolidated into (**Figures 4A–F**). For each organ, at least 50 structures per animal were measured from three random fields (n = 6 per group) using ImageJ.

Heart (**Figure 4A**): WT−SHAM mice exhibited a significant increase in mean cardiomyocyte cross−sectional area compared to WT−CON (458.1 ± 34.46 µm² vs. 251.9 ± 25.01 µm², P < 0.001). RYGB surgery reduced this area in WT−RYGB mice to levels comparable to WT−CON (P = 0.979). All KO groups showed larger cardiomyocyte areas than their WT counterparts (P < 0.001).

Liver (**Figure 4B**): H&E-stained liver sections (**Supplementary Figure 4**) showed corresponding morphological changes (e.g., hepatocyte ballooning and inflammatory cell foci). Hepatic steatosis was assessed by Oil Red O staining (quantified as lipid−positive area percentage). WT−SHAM mice showed a marked increase in lipid area compared to WT−CON (1.421% ± 0.2172% vs. 0.8000% ± 0.06621%, P < 0.001). RYGB significantly reduced lipid accumulation in WT−RYGB mice. KO groups consistently displayed higher steatosis than WT groups across all conditions (CON: P < 0.01; RYGB: P < 0.05; SHAM: P < 0.05).

Pancreas (**Supplementary Figure 5**): H&E−stained pancreatic sections showed genotype−dependent structural changes. SHAM−operated mice exhibited mild acinar disorganization, loss of basal polarity, and abnormal nuclear−to−cytoplasmic ratios. RYGB improved cytoarchitecture in both genotypes, with restored acinar organization. WT mice maintained orderly lobulation and intact ductal networks, whereas KO mice showed progressive acinar derangement across all groups.

Kidney (**Figure 4C**): Glomerular cross−sectional area was measured. WT−SHAM mice had larger glomerular area than WT−CON (3893 ± 200.3 µm² vs. 2144 ± 199.1 µm², P < 0.001). RYGB surgery reduced glomerular area in both genotypes. KO−CON mice showed larger glomerular area than WT−CON (P = 0.002), and KO−SHAM had larger area than WT−SHAM (P < 0.001). After RYGB, the difference between genotypes was no longer significant (KO−RYGB vs. WT−RYGB: P = 0.056). No overt glomerular or tubular damage was observed in any group; H&E staining is insufficient to assess finer structures such as the brush border.

Inguinal adipose tissue (**Figure 4D**): RYGB induced a marked reduction in adipocyte area in both genotypes compared to SHAM (both P < 0.001). KO−CON adipocytes were significantly larger than WT−CON (P = 0.002), and KO−SHAM exhibited larger adipocytes than WT−SHAM (P = 0.016). Notably, RYGB eliminated this genetic divergence, with KO−RYGB and WT−RYGB showing equivalent adipocyte dimensions (P = 0.934).

Skeletal muscle (**Figure 4E**): SHAM−operated mice exhibited marked myofiber atrophy compared to CON (P < 0.001). RYGB substantially reversed these atrophic changes in both genotypes (WT−RYGB vs. WT−SHAM: P = 0.002; KO−RYGB vs. KO−SHAM: P < 0.001). No significant genotype differences were observed (P > 0.05).

All data are shown as mean ± SD (n = 6 per group). Scale bars = 50 µm for all panels. For detailed statistical comparisons, see the figure legend of **Figure 4**.

### 3.5 RYGB surgery elevates IGFBP-1-dependent components of the insulin signaling axis in circulation

**Figure 5:**
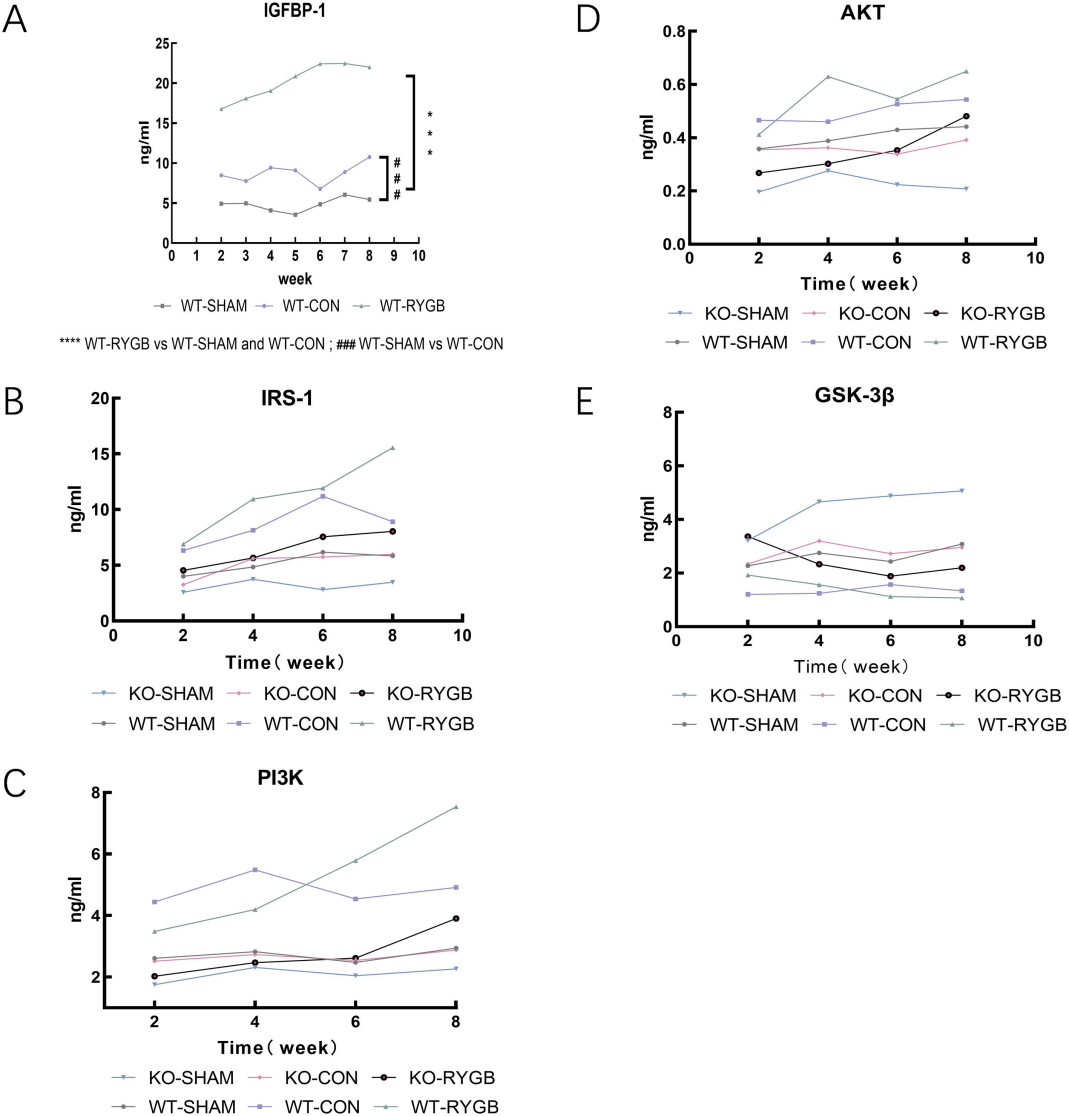
Serum levels of IGFBP-1, IRS−1, PI3K, AKT and GSK−3β in WT and IGFBP−1 KO mice at 8 weeks post−surgery. Plasma concentrations of **(A)** IGFBP-1, **(B)** IRS-1, **(C)** PI3K, **(D)** AKT, and **(E)** GSK-3β measured by ELISA. Data are shown as mean ± SD (n = 6 per group). Statistical analyses: one-way ANOVA with LSD post-hoc test (within each genotype) and independent t-tests (WT vs. KO within the same surgical condition). *P < 0.05, **P < 0.01, ***P < 0.001.

Molecular analyses demonstrated that RYGB surgery dynamically modulates plasma IGFBP-1 kinetics and downstream insulin signaling pathways in type 2 diabetic mice. Postoperative plasma IGFBP-1 levels in WT-RYGB mice were significantly elevated compared to WT-SHAM and WT-CON groups at week 2 (F = 179.8, P < 0.001, **Figure 5A**), exhibiting a sustained upward trajectory before plateauing at steady-state concentrations. Notably, WT-SHAM mice maintained suppressed IGFBP-1 levels compared with WT-CON (P < 0.001). As expected, IGFBP-1 was undetectable in the serum of KO mice, confirming the successful knockout.

In WT mice, RYGB surgery induced a significant and coordinated elevation of circulating IRS-1, PI3K, and AKT levels, along with suppression of GSK-3β, compared to WT-SHAM and WT-CON controls (**Figures 5B–E**, all P < 0.001). Critically, this surgery-induced coordinated response was completely absent in IGFBP-1 KO mice. The serum levels of IRS-1, PI3K, and AKT in KO-RYGB mice remained similar to those in KO-SHAM and KO-CON groups, and GSK-3β remained elevated. This genotype-dependent difference provides strong genetic evidence that IGFBP-1 is necessary for the systemic upregulation of this signaling axis following RYGB.

### 3.6 Hepato-centric IGFBP-1 transcriptional regulation by RYGB surgery

**Figure 6:**
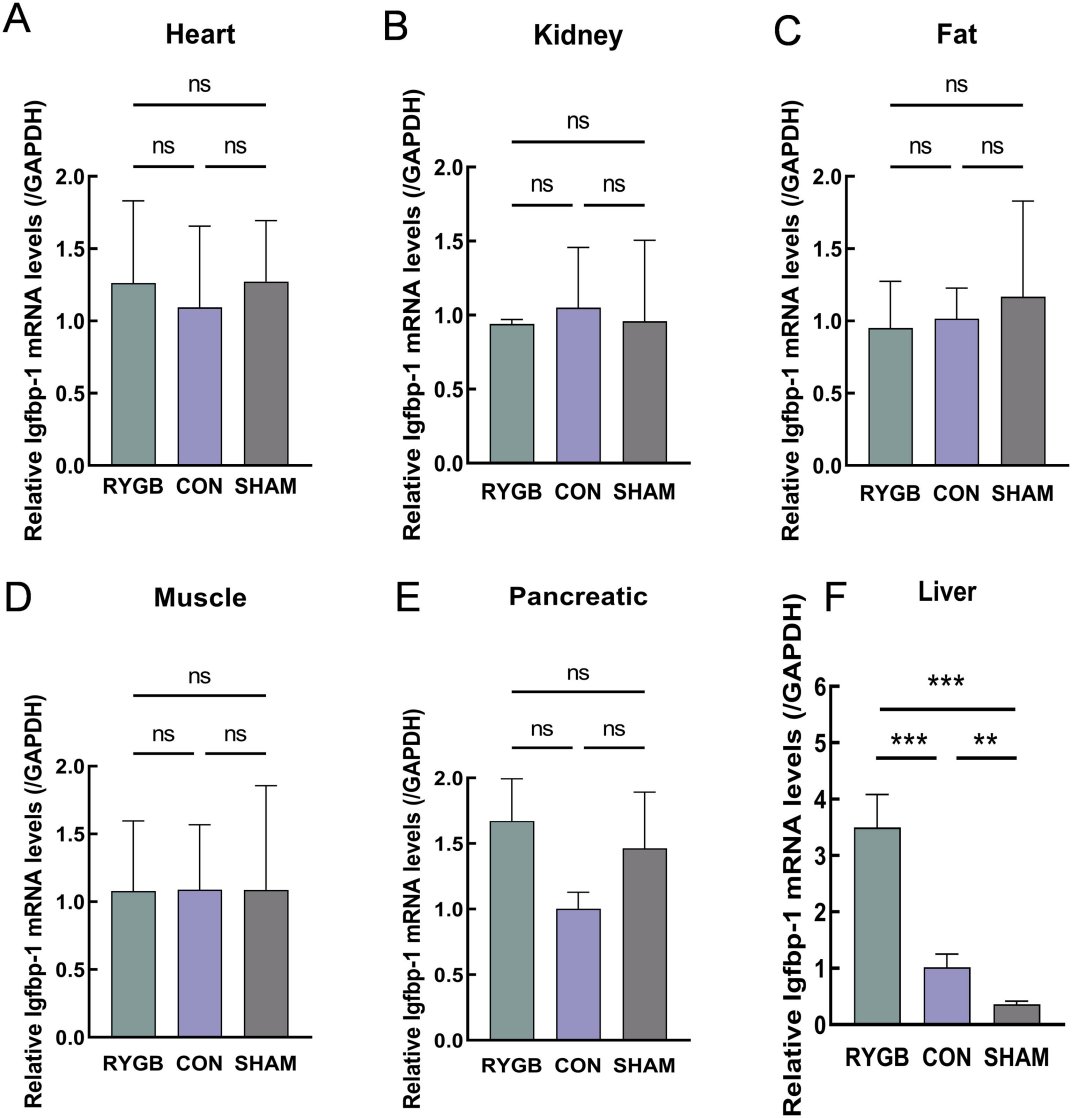
Tissue−specific expression of IGFBP−1 mRNA in WT mice. Relative mRNA expression of IGFBP-1 in heart, kidney, fat, skeletal muscle, pancreas, and liver of WT mice at 8 weeks post-surgery, measured by RT-PCR (GAPDH as internal control, 2^-ΔΔCt method). Data are shown as mean ± SD (n = 3 per group). One-way ANOVA followed by Tukey’s post-hoc test was used for each tissue. No significant differences were observed in peripheral tissues (heart, kidney, fat, muscle, pancreas; all P > 0.05). In contrast, hepatic IGFBP-1 expression showed significant differences among groups (F = 188.6, P < 0.001).

Consistent with established tissue distribution, IGFBP-1 synthesis occurred predominantly in the liver. qPCR analysis revealed that peripheral tissues (cardiac (27, 28), hepatic (29), pancreatic (30), renal (29), skeletal muscle (31, 32), and adipose (33)), IGFBP-1 synthesis occurs predominantly in the liver (29, 34, 35). Quantitative RT-PCR analysis (GAPDH-normalized, 2^-ΔΔCt method) revealed profound tissue-specific regulation: Peripheral tissues including heart (F = 0.3808, P = 0.687), pancreas (F = 0.7734, P = 0.472), kidney (F = 0.1128, P = 0.894), and skeletal muscle (F = 0.1332, P = 0.876) exhibited no significant IGFBP-1 mRNA alterations across experimental groups. In striking contrast, hepatic tissue demonstrated RYGB-specific transcriptional upregulation, with WT-RYGB mice showing upregulation compared with WT-SHAM and WT-CON controls (F = 188.6, P < 0.001, **Figure 6**). Notably, WT-SHAM mice exhibited 38.2% reduced expression relative to WT-CON (P < 0.01), confirming obesity-induced suppression of hepatic IGFBP-1 transcription. These results establish the liver as the exclusive site of RYGB-mediated IGFBP-1 induction, explaining the surgery-dependent plasma IGFBP-1 elevation observed in Section 5.

### 3.7 Hepatocentric IGFBP-1 orchestrates insulin signaling reprogramming post-RYGB

**Figure 7:**
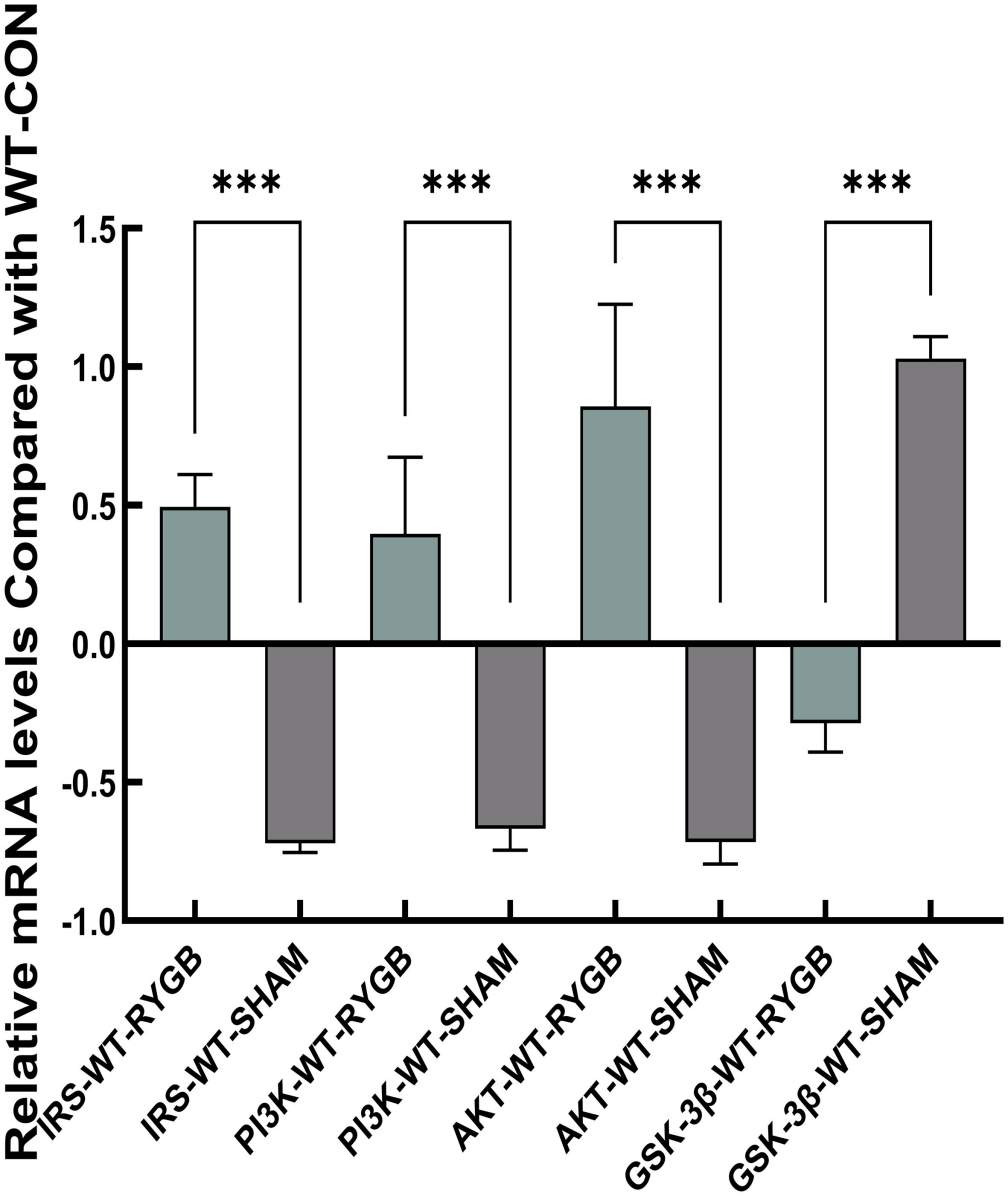
Hepatic mRNA expression of insulin signaling components in WT mice. Relative mRNA expression of IRS-1, PI3K, AKT, and GSK-3β in the liver of WT mice at 8 weeks post-surgery, measured by qRT-PCR (GAPDH as internal control, 2^-ΔΔCt method). Data are shown as mean ± SD (n = 6 per group). Comparisons between WT-RYGB and WT-SHAM were performed using independent t-tests. ***P < 0.001 for all comparisons (IRS-1, PI3K, AKT, and GSK-3β).

Building on the liver-specific IGFBP-1 induction (Section 6), qPCR analysis of hepatic insulin signaling components revealed RYGB-mediated transcriptional reprogramming that mirrored prior protein-level findings (Section 5). In WT-RYGB mice compared with WT-SHAM controls, we observed coordinated upregulation of insulin signaling effectors: IGFBP-1 expression increased (F = 188.6, P < 0.001, **Figure 7**), IRS-1 expression surged (t = 37.38, P < 0.001), PI3K expression increased (t = 13.62, P < 0.001), and AKT expression increased (t = 16.21, P < 0.001). This triad increase signifies enhanced insulin signal transduction, potentially regulating core metabolic functions including energy homeostasis and insulin sensitivity. Conversely, GSK-3β expression was suppressed in RYGB compared with SHAM (t = 84.64, P < 0.001), aligning with its role as a glycogenolysis gatekeeper.

## 4 Discussion

Our multi-dimensional phenotypic analysis consistently underscores the pivotal role of IGFBP-1 in maintaining metabolic homeostasis. IGFBP-1 knockout mice exhibited inherent metabolic susceptibility, displaying higher body weight, more severe fasting hyperglycemia, and elevated HOMA-IR compared to WT mice. Histopathologically, IGFBP-1 deficiency exacerbated diabetes-associated multi-organ damage: cardiomyocyte hypertrophy, hepatic steatosis, increased adipocyte size, and glomerular hypertrophy. RYGB surgery drove significant benefits independent of IGFBP-1, as seen in KO-RYGB mice compared to KO-SHAM controls. However, recovery in KO mice plateaued at partial amelioration. In contrast, WT-RYGB mice achieved full restoration: normalized fasting glucose, HOMA-IR, and near-complete reversal of multi-organ pathology. Therefore, IGFBP-1 is not the sole initiator of post-surgical improvement but emerges as an essential determinant for attaining the upper limit of therapeutic efficacy.

The interpretation of circulating signaling proteins deserves comment. While serum total protein levels do not directly quantify tissue-specific phosphorylation, the coordinated, genotype-specific elevation of this entire panel of signaling molecules in plasma represents a biologically significant finding. It suggests a systemic alteration in the synthesis, secretion, or clearance of these proteins, reflecting a whole-body recalibration of the metabolic signaling axis in response to RYGB, contingent upon IGFBP-1. The PI3K/Akt/GSK-3β signaling axis plays an indispensable role in mediating insulin’s metabolic actions (36, 37). Genetic ablation or mutation of key components results in severe insulin resistance (36, 37). Elevated GSK-3β activity in type 2 diabetic muscle further underscores the relevance of this signaling axis (38, 39). We acknowledge the limitations of this approach. The absence of KO tissue mRNA data limits our ability to claim that the liver transcriptional response is IGFBP-1-dependent. However, the serum genotype comparison already demonstrates that the systemic protein response requires IGFBP-1. Direct measurement of IRS-1 and AKT phosphorylation in insulin target tissues would provide definitive proof of pathway elevation, and we have noted this as an important direction for future studies.

Consistent with established tissue distribution, IGFBP-1 synthesis occurs predominantly in the liver. qPCR analysis revealed that peripheral tissues including heart, pancreas, kidney, skeletal muscle, and adipose exhibited no significant IGFBP-1 mRNA alterations across experimental groups. In striking contrast, hepatic tissue demonstrated RYGB-specific transcriptional upregulation, with WT-RYGB mice showing upregulation compared with WT-SHAM and WT-CON controls. This establishes the liver as the exclusive source of the surgery-induced IGFBP-1 elevation observed in the circulation. Given that IGFBP-1 is a secreted protein, the elevated circulating levels are positioned to act systemically on peripheral tissues. However, our current data do not distinguish between systemic endocrine action versus local paracrine/autocrine effects at peripheral sites. IGFBP-1 contains a conserved RGD (Arg-Gly-Asp) domain, which enables direct interaction with integrin receptors on target cells. This raises the intriguing possibility that circulating IGFBP-1 may directly signal through integrin pathways independent of IGF-1, thereby modulating insulin sensitivity in peripheral tissues such as skeletal muscle, adipose tissue, and liver. This would represent a distinct mechanism from the classical IGF-1-binding function of IGFBPs. Alternatively, IGFBP-1 may regulate IGF-1 bioavailability, indirectly modulating insulin signaling. Future studies employing tissue-specific IGFBP-1 receptor knockout models or conditional overexpression systems will be required to definitively distinguish between systemic and local actions.

The restoration of glucose homeostasis after RYGB is multifaceted. Established mechanisms include enhanced secretion of GLP-1, alterations in bile acid composition, and shifts in gut microbiota (10–12). Our discovery of the IGFBP-1-dependent pathway does not negate these contributors but reveals a significant, liver-centric endocrine arm. This positions IGFBP-1 as a key mediator that potentially integrates with signals from the altered gut environment.

Our findings are consistent with and extend recent human studies. Our prior clinical work in Chinese patients with obesity demonstrated significant elevation of serum IGFBP-1 six months after RYGB, accompanied by reduced HOMA-IR (13). This observation has been corroborated by other groups: recent studies have reported that elevated IGFBP-1 correlates with improved glycemic control and enhanced insulin sensitivity after bariatric surgery, including sleeve gastrectomy and RYGB. Notably, a longitudinal study showed that early postoperative IGFBP-1 elevation predicted sustained diabetes remission at 2 years, suggesting potential utility as an early response marker (40). However, mechanistic validation in human tissues remains challenging. Our current study provides the first genetic evidence in a mammalian model that IGFBP-1 is not merely a biomarker, but a functionally necessary mediator of surgical recovery. This transition from biomarker to mediator has important implications for how we interpret and potentially leverage IGFBP-1 in clinical settings.

The identification of IGFBP-1 as a critical mediator of RYGB’s full metabolic benefits raises several clinical considerations. First, pre-surgical IGFBP-1 levels may serve as a predictive biomarker for surgical outcomes. Second, IGFBP-1 or its downstream effectors could represent novel therapeutic targets for patients who are not candidates for surgery or who experience incomplete remission. However, our current understanding is limited, and significant further investigation is required before these possibilities can be translated to clinical practice.

We acknowledge several important limitations. First, while the HFD + low-dose STZ model is widely accepted for T2DM studies, it does not fully recapitulate human polygenic T2DM, and the potential for a type 1-like component cannot be completely excluded. Second, the lack of pair-fed controls represents a limitation of this study. RYGB surgery induces significant weight loss and reduced caloric intake, both of which independently improve insulin sensitivity. Without pair-fed sham-operated animals, we cannot definitively dissociate the specific metabolic effects of RYGB from those secondary to caloric restriction. However, our study design of comparing WT and KO mice subjected to identical surgical procedures allows us to assess the genotype-dependent contribution to the surgical outcome, as any caloric intake differences between genotypes would be expected to minimize rather than enhance the observed genotype-specific effects. Nevertheless, future studies incorporating pair-fed controls will be valuable to isolate IGFBP-1-mediated effects from generalized weight-loss benefits. Third, our mechanistic conclusions are based primarily on serum protein levels and tissue mRNA expression. We acknowledge that total plasma levels of IRS−1, PI3K, AKT, and GSK−3β do not directly reflect their phosphorylation status. While circulating protein levels do not report on tissue−specific phosphorylation, the coordinated, genotype−specific elevation of this entire panel of signaling molecules in plasma represents a biologically significant finding, suggesting a systemic alteration in the synthesis, secretion, or clearance of these proteins in response to RYGB, contingent upon IGFBP−1. While we used plasma ELISAs for consistency with our prior clinical study, the observed IGFBP−1−dependent increases in circulating IRS−1, PI3K, and AKT were corroborated by hepatic qPCR data. However, direct evidence of pathway activation is required to confirm causality. Our conclusions are therefore framed as IGFBP−1 being necessary for the upregulation of this signaling axis, not as definitive proof of its activation. Future phospho−specific assays are needed. Fourth, the histological morphometry, although quantified, involved relatively small sample sizes and kidney histology was assessed by H&E staining only, which is insufficient to evaluate tubular integrity.

## 5 Conclusion

This study demonstrates that RYGB surgery ameliorates obesity and diabetic pathophysiology. While surgery confers metabolic benefits independent of genotype, the attainment of complete metabolic normalization, defined by restoration of normoglycemia, full insulin sensitivity (normalized HOMA-IR), and reversal of multi-organ pathology, is critically dependent on IGFBP-1. Mechanistically, IGFBP-1 is necessary for the upregulation of the IRS-1/PI3K/AKT/GSK-3β signaling axis observed after RYGB, which underlies the restoration of systemic insulin sensitivity. Genetic ablation of IGFBP-1 attenuated this pathway upregulation and resulted in an incomplete recovery phenotype despite surgical intervention. Thus, IGFBP-1 transitions from a nutritional biomarker to an essential mediator of the full therapeutic efficacy of metabolic surgery. Future studies employing phospho-specific assays in insulin target tissues will be valuable to directly confirm the elevation status of this signaling cascade downstream of IGFBP-1. In addition, investigating whether circulating IGFBP-1 acts primarily through systemic endocrine mechanisms, local tissue-specific actions, or both, represents another important avenue for future research.

## Data Availability

The original contributions presented in the study are included in the article/**Supplementary Material**, further inquiries can be directed to the corresponding author/s.
