# Supplementary figures and images for: IGFBP-1 is associated with IRS signaling upregulation and contributes to metabolic recovery post-Roux-en-Y bypass
Source: Front Endocrinol (Lausanne). 2026 Jul 16;17:1859266. doi: 10.3389/fendo.2026.1859266 (PMC13422207; doi:10.3389/fendo.2026.1859266)

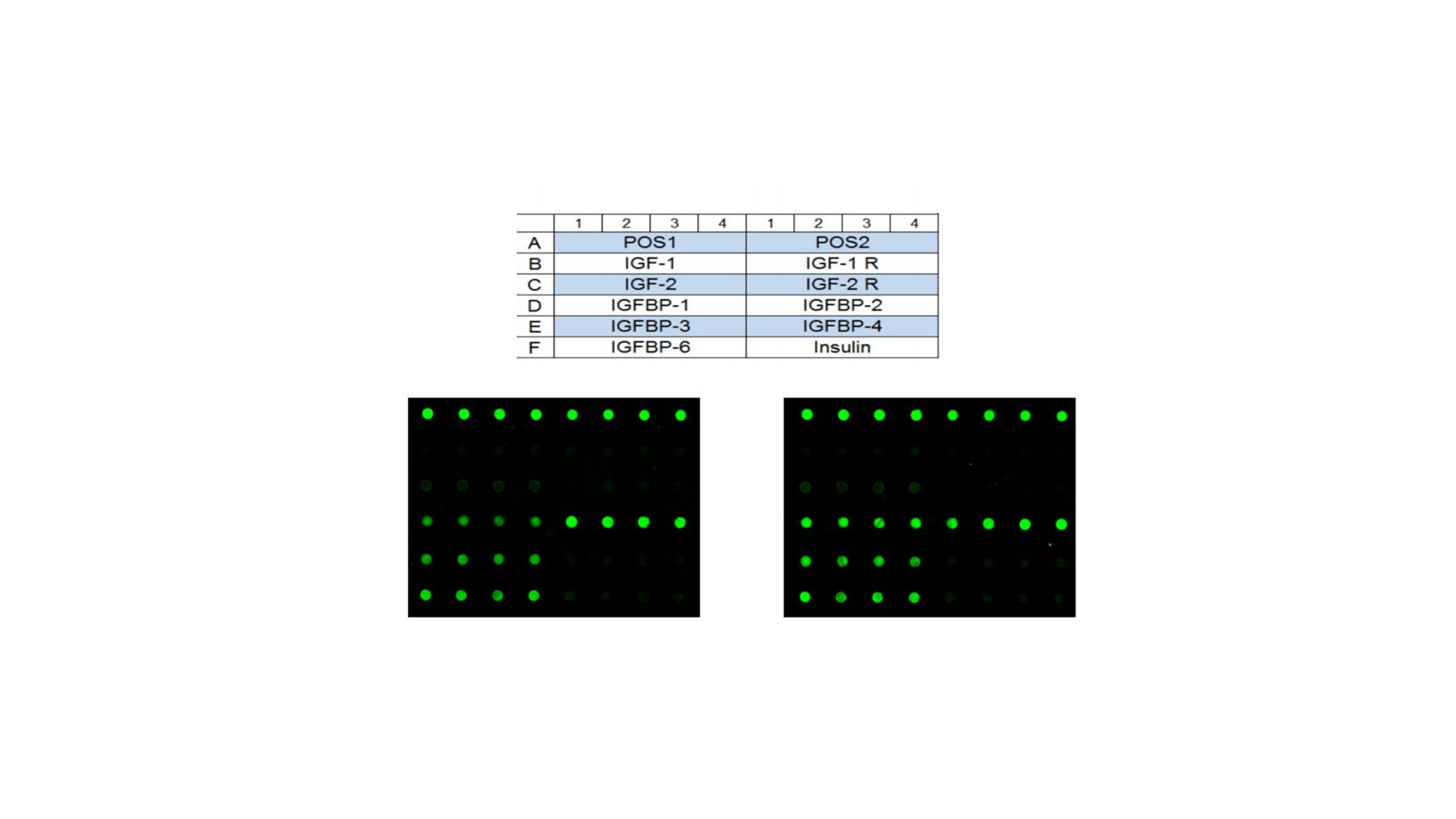

Supplement: Supplementary file 1 [file Image1.tif]

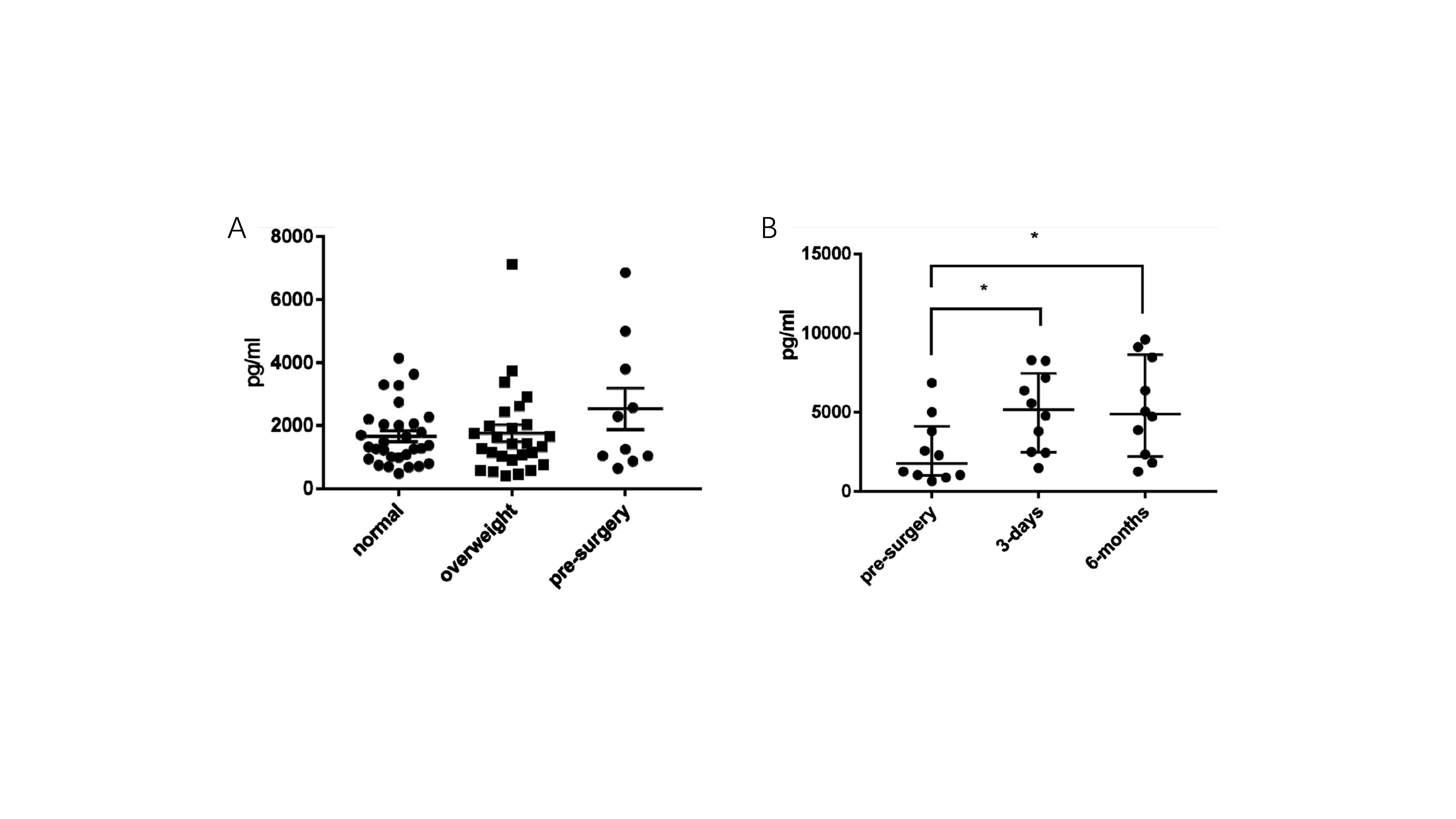

Supplement: Supplementary file 2 [file Image2.tif]

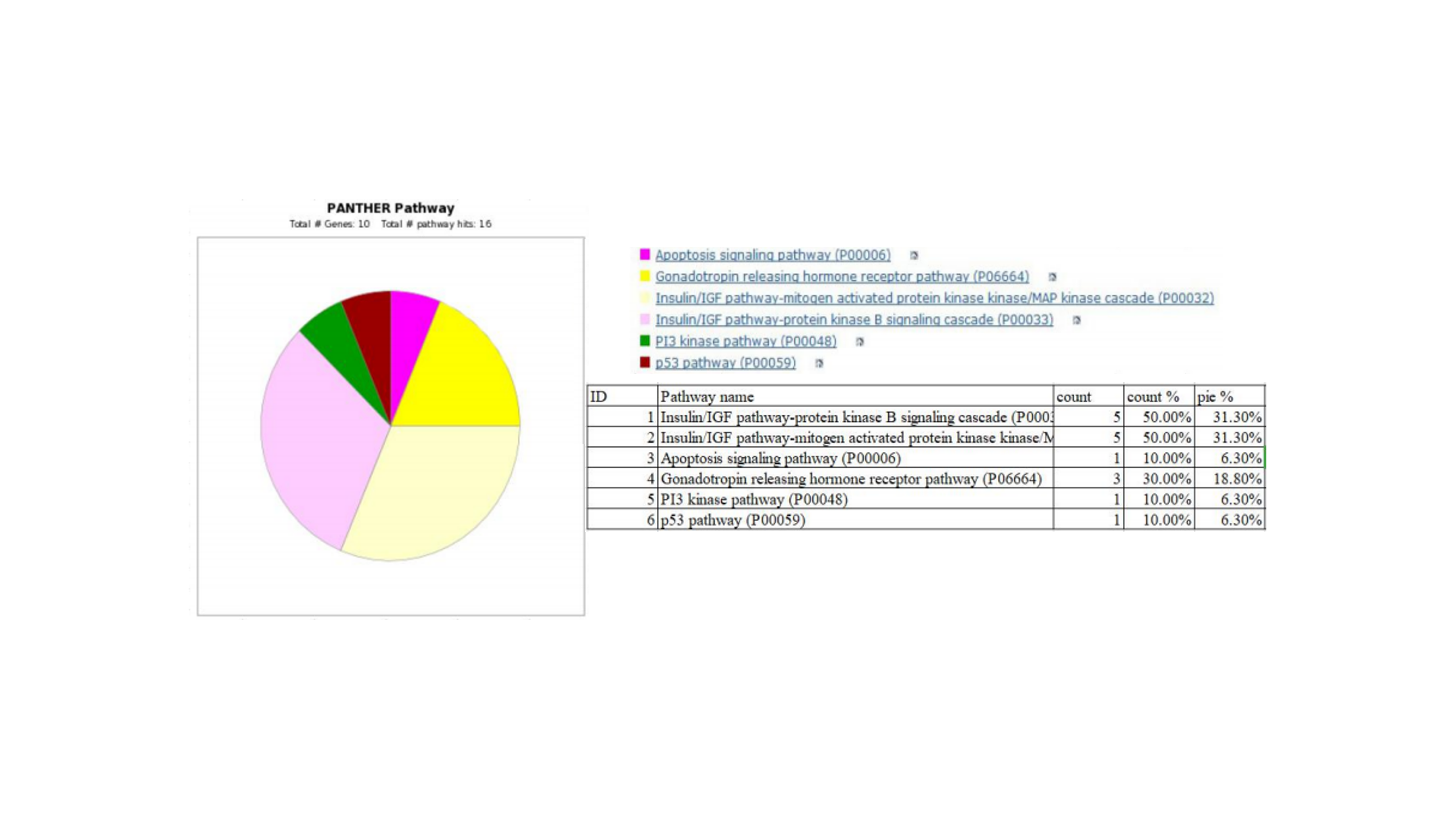

Supplement: Supplementary file 3 [file Image3.tif]

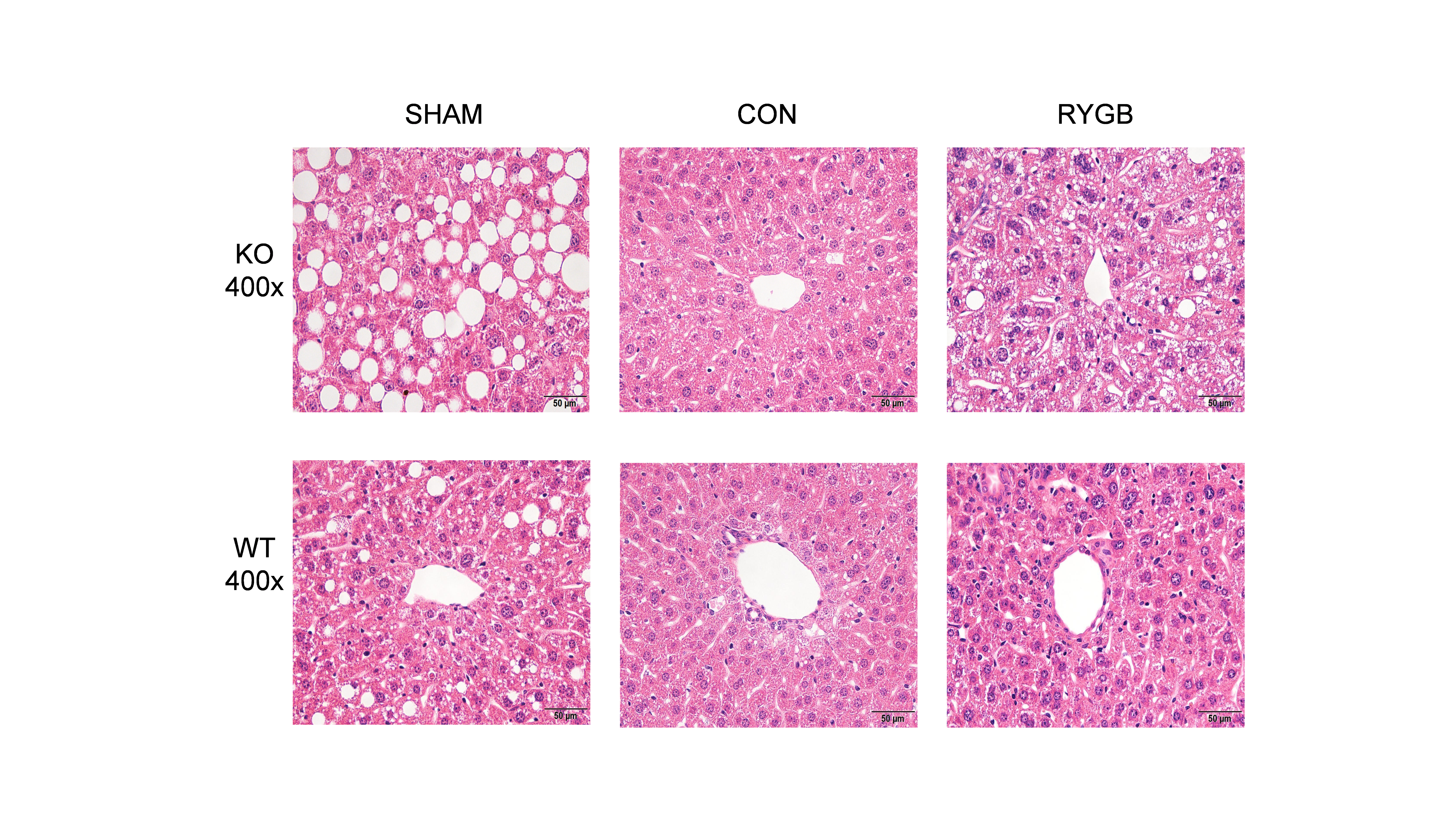

Supplement: Supplementary file 4 [file Image4.tif]

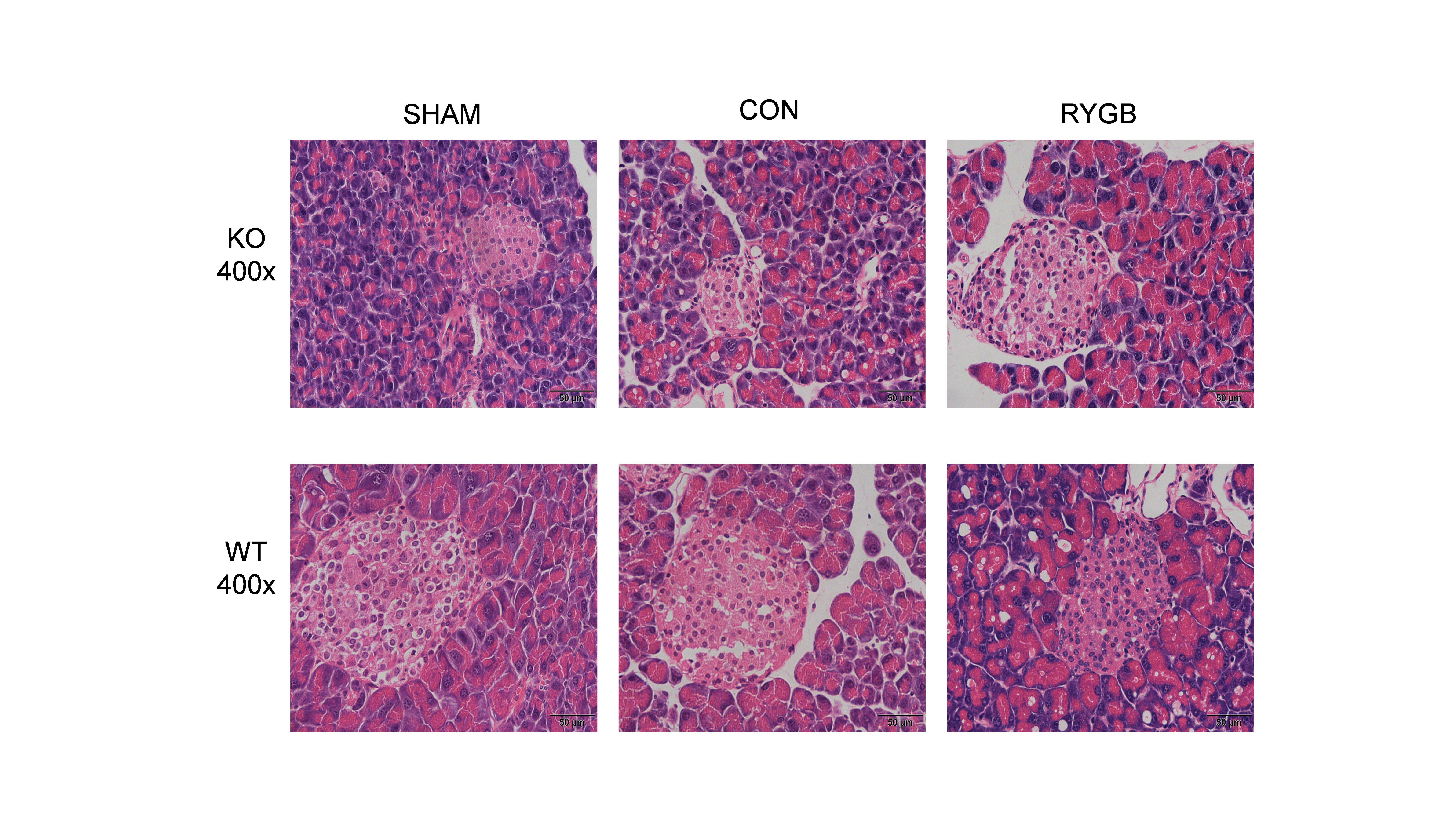

Supplement: Supplementary file 5 [file Image5.tif]
